# Cost consequence analysis of waiting for lumbar disc herniation surgery

**DOI:** 10.1038/s41598-023-31029-5

**Published:** 2023-03-18

**Authors:** Charlotte Dandurand, Mohammad Sadegh Mashayekhi, Greg McIntosh, Supriya Singh, Jerome Paquet, Hasaan Chaudhry, Edward Abraham, Christopher S. Bailey, Michael H. Weber, Michael G. Johnson, Andrew Nataraj, Najmedden Attabib, Adrienne Kelly, Hamilton Hall, Y. Raja Rampersaud, Neil Manson, Philippe Phan, Ken Thomas, Charles Fisher, Raphaele Charest-Morin, Alex Soroceanu, Bernard LaRue, Nicolas Dea

**Affiliations:** 1 Combined Neurosurgical and Orthopedic Spine Program, Department of Orthopedics Surgery, University of British Columbia, Blusson Spinal Cord Center, 6th Floor, 818 West 10th Avenue, Vancouver, BC V5Z 1M9 Canada; 2 Canadian Spine Outcomes and Research Network, Markdale, ON Canada; 3 Centre de Recherche CHU de Quebec, CHU de Quebec-Universite Laval, Quebec City, QC Canada; 4 Division of Orthopaedic Surgery, Zone 2, Horizon Health Network, Canada East Spine Centre, Saint John, NB Canada; 5 London Health Science Centre Combined Neurosurgical and Orthopaedic Spine Program, Schulich School of Medicine, Western University, London, ON Canada; 6 Department of Surgery, Division of Orthopaedics, Montreal General Hospital, McGill University, Montreal, QC Canada; 7 Department of Surgery, Section of Orthopedics and Neurosurgery, University of Manitoba, Winnipeg, MB Canada; 8 Division of Neurosurgery, University of Alberta, Edmonton, AB Canada; 9 Division of Neurosurgery, Zone 2, Horizon Health Network, Canada East Spine Centre, Saint John, NB Canada; 10 Sault Area Hospital, Northern Ontario School of Medicine, Sault Ste Marie, ON Canada; 11 Department of Surgery, University of Toronto, Toronto, ON Canada; 12 Schroeder Arthritis Institute, Krembil Research Institute, University Health Network, Orthopaedics, Department of Surgery, University of Toronto, Toronto, ON Canada; 13 Division of Orthopaedic Surgery, University of Ottawa, Ottawa Hospital, Ottawa, ON Canada; 14 University of Calgary Spine Program, University of Calgary, Calgary, AB Canada; 15 Départment de chirurgie, Faculté de Médecine et des Sciences de la Santé, Université de Sherbrooke, Sherbrooke, QC Canada; 16 Sunnybrook Hospital, University of Toronto, Toronto, ON Canada

**Keywords:** Health care, Medical research

## Abstract

The economic repercussions of waiting for lumbar disc surgery have not been well studied. The primary goal of this study was to perform a cost-consequence analysis of patients receiving early vs late surgery for symptomatic disc herniation from a societal perspective. Secondarily, we compared patient factors and patient-reported outcomes. This is a retrospective analysis of prospectively collected data from the CSORN registry. A cost-consequence analysis was performed where direct and indirect costs were compared, and different outcomes were listed separately. Comparisons were made on an observational cohort of patients receiving surgery less than 60 days after consent (short wait) or 60 days or more after consent (long wait). This study included 493 patients with surgery between January 2015 and October 2021 with 272 patients (55.2%) in the short wait group and 221 patients (44.8%) classified as long wait. There was no difference in proportions of patients who returned to work at 3 and 12-months. Time from surgery to return to work was similar between both groups (34.0 vs 34.9 days, p = 0.804). Time from consent to return to work was longer in the longer wait group corresponding to an additional $11,753.10 mean indirect cost per patient. The short wait group showed increased healthcare usage at 3 months with more emergency department visits (52.6% vs 25.0%, p < 0.032), more physiotherapy (84.6% vs 72.0%, p < 0.001) and more MRI (65.2% vs 41.4%, p < 0.043). This corresponded to an additional direct cost of $518.21 per patient. Secondarily, the short wait group had higher baseline NRS leg, ODI, and lower EQ5D and PCS. The long wait group had more patients with symptoms over 2 years duration (57.6% vs 34.1%, p < 0.001). A higher proportion of patients reached MCID in terms of NRS leg pain at 3-month follow up in the short wait group (84.0% vs 75.9%, p < 0.040). This cost-consequence analysis of an observational cohort showed decreased costs associated with early surgery of $11,234.89 per patient when compared to late surgery for lumbar disc herniation. The early surgery group had more severe symptoms with higher healthcare utilization. This is counterbalanced by the additional productivity loss in the long wait group, which likely have a more chronic disease. From a societal economic perspective, early surgery seems beneficial and should be promoted.

## Introduction

Lumbar disc herniation is a common cause of back and leg symptoms^1^. While non-operative management is often initially proposed, some fail to improve and eventually undergo lumbar discectomy, one of the most common surgical procedures performed in Canada^2,3^. As disc herniation is a very common pathology with an available low risk procedure, discectomy represents 70–90% of all outpatient spinal surgery ^2,4^. Several studies have identified discectomy to be cost effective compared to non-surgical management^2,5–8^. A cost-effectiveness analysis revealed increased workers’ earnings of $1925 (95% confidence interval $1121–$2728) with fewer missed workdays for patients receiving surgery compared to non-surgical treatment^6^.

Many patients deemed surgical end up waiting several weeks to months for surgery, delaying return to their prior level of functioning^3,9–13^. In Canada, access to elective lumbar discectomy is managed through surgical waitlists that are often maintained by each surgeon’s offices, which can create variation in wait times. For reference, 43% of Canadian spine surgeons have noted a wait of over 6 months for surgery^14^. However, many different healthcare systems worldwide are faced with the same reality of variation in wait times. The cause of this wait time is multifactorial such as clinical severity, limited access to the operating room and considerable variation in institutional and surgeon practices^15^. In a single healthcare payer system, wait times for these elective procedures can be considerable and the economic consequences of waiting are poorly understood, especially when considering indirect costs from a societal perspective. Replacement wages are responsible for up to 80–90% of total societal costs of low back pain^16,17^. Therefore, it is crucial to include indirect costs to better understand the economic impact of the timing of such a commonly performed surgical procedure.

This study aims to provide meaningful new comparative evidence to decision makers who plan healthcare services. The main goal of this study was to perform a cost-consequence analysis of direct and indirect cost differences of early vs late surgery for symptomatic lumbar disc herniation. Secondarily, we report on patient reported outcome measures (PROMs) and compare the proportions of patients who reached meaningful clinically important difference (MCID) between early and late surgery.

## Methods

### Study design

A cost-consequence analysis was performed where direct and indirect costs were compared, and different outcomes were listed separately. This study used a societal perspective. This is a retrospective analysis of prospectively collected data from the multi-center Canadian Spine Outcomes and Research Network (CSORN), a national registry that includes over 60 neurosurgical and orthopedic spine surgeons from 18 academic and community hospitals across Canada. All research was performed in accordance with relevant guidelines/regulations. Research was performed in accordance with the *Declaration of Helsinski*. The *UBC Clinical Research Ethics Board (CREB*) approved the study and waived the need of informed consent at this study was retrospective. At all sites, standardized data collection is performed in the preoperative and post-operative periods at pre-specified time points. For this study, clinical data used was collected at time of enrolment and 3-months. For inclusion in the study, patients had to have time to return to work available at 3 months. Return to work at 12-month follow-up was also collected. Patients were enrolled between January 2015 and October 2021. Patient inclusion criteria were: (1) 18 years old or more; (2) surgically treated for lumbar disc herniation; (3) actively working or employed but not working at time of enrolment; (4) no previous surgery; (5) not involved in litigation or workers’ compensation claims; (6) treated electively; and (7) had minimum 3 months follow-up data including time to return to work available.

### Data collection

Baseline was defined as the date of consent. Baseline patient variables collected were age, sex, number of comorbidities, body mass index (BMI), marital status, living status, education level, exercise status, medication usage, smoking status, symptoms duration (more or less than 2 years) and surgical date. PROMs and health care utilization were collected at baseline and at 3-months post-operatively^18^. PROMs collected were numeric rating scale for back pain (NRS back), numeric rating scale for leg pain (NRS leg), Oswestry Disability Index (ODI), Euroquol EQ5D, and the Physical Component Score (PCS) and Mental Component Score (MCS) of the SF12. Healthcare utilization was measured by healthcare professional visits and diagnostic imaging performed. We made the assumption that healthcare utilization in the postoperative period was similar between the short wait and long wait groups and any difference occurred during the period before surgery. Healthcare utilization included physician visits (visits to emergency department, surgeons from non-spine specialty, non-surgical physicians, pain specialists and/or family doctors) and other healthcare professionals (naturopathy, chiropractic, physiotherapy, occupational therapy, massage therapy, acupuncture and/or personal training). The use of diagnostic imaging included use of x rays, CT scan, magnetic resonance imaging (MRI), electromyography (EMG), bone scan, injections using fluoroscopy and injections using other imaging techniques. Working status and time to return to work was collected at 3-months and 12-months follow-up.

### Outcome

The consolidated costs included direct costs incurred by healthcare utilization and the indirect costs included the economic value of productivity loss related to time off work. Comparisons were made between those receiving surgery less than 60 days after consent (short wait) and 60 days or more after consent (long wait). This cut-off time-point of 60 days has been commonly used in previous literature^19,20^. Patients’ assignment to the short or long wait groups were not randomized and were based on the treating surgeon’s individual practice. The timing for surgery was based notably on available resources, waitlists and clinical severity. The primary outcome was defined as the cost difference between long wait and short wait. Direct expenses related to the surgery were assumed to be the same between the two groups.

Time off work was assumed to be from time of consent and was calculated in days from (1) the date of consent (baseline) to 3 months, (2) the date of consent (baseline) to 12 months follow-up, (3) from surgery to 3 months, and (4) from surgery to 12 months follow-up. Patients were asked to report their exact return to work date.

### Expenditure data

Unit costs were inflated to 2021 using consumer price index calculator made publicly available by Statistics Canada and Bank of Canada^21^. Healthcare utilization costs were extracted from the Ontario Health Insurance Plan (OHIP) claims history database and Ontario Case Costing Initiative^22–24^. Differing costs between groups were extracted and are presented in Table 1. The cost difference in each healthcare utilization between groups was divided by the total number of patients in the group with the excess in healthcare utilization to obtain the additional healthcare usage direct cost per patient. The human-capital method was used to take the patient’s perspective and count the time not worked. This method was favored to the friction method which takes the employer’s perspective and only counts costs until another employee takes over the patient’s work^25^. Work replacement data was not available. Wages were used as a proxy measure of employee output. The amount of missed work was multiplied by the Canadian national average wage. Using Canada Income Statistics, the 2021 average yearly salary for the fully employed was estimated to be ~ $54,630. We assumed 2 weeks leave as per the average Canadian^26^ and removed weekend days to calculate a daily pay of 217.65$.

**Table 1:** Differing direct and indirect costs.

	Cost (2021* CAD)	References
Consultation with emergency physician	$215.00	Ontario Health Insurance Plan Claims History and Ontario Case Costing Initiative^22–24^
MRI one spinal segment	$1016.10	Ontario Health Insurance Plan Claims History and Ontario Case Costing Initiative^22–24^
Physiotherapy session (average)	$100.00	Ontario Professional Fees Guideline^25,26^
Daily average Canadian pay 2021	$217.65	Canada Income Statistics

*Costs were inflated to 2021 using consumer price index calculator via Statistics Canada and Bank of Canada.

### Statistical analysis

Continuous data were summarized using means and standard deviations and compared using Student’s T-tests. Categorical data are expressed as numbers and percentages, compared using Chi-Square tests. The minimum clinically important difference (MCID) was defined as ≥ 30% improvement from unadjusted baseline NRS leg and NRS back to unadjusted 3 months^27,28^. Other MCID were defined as: ODI, 15 points; EQ-5D, 0.4; PCS, 7; and MCS, 7^29–31^. The proportion of patients reaching MCID was compared between patients who returned to work at 3 months or not and between short and long wait groups. Also, changes in PROMs at 3-months were adjusted for baseline score. A p value < 0.05 was considered statistically significant. Analyses were performed with IBM SPSS for Windows release 28.

## Results

### Patient characteristics

**Figure 1:**
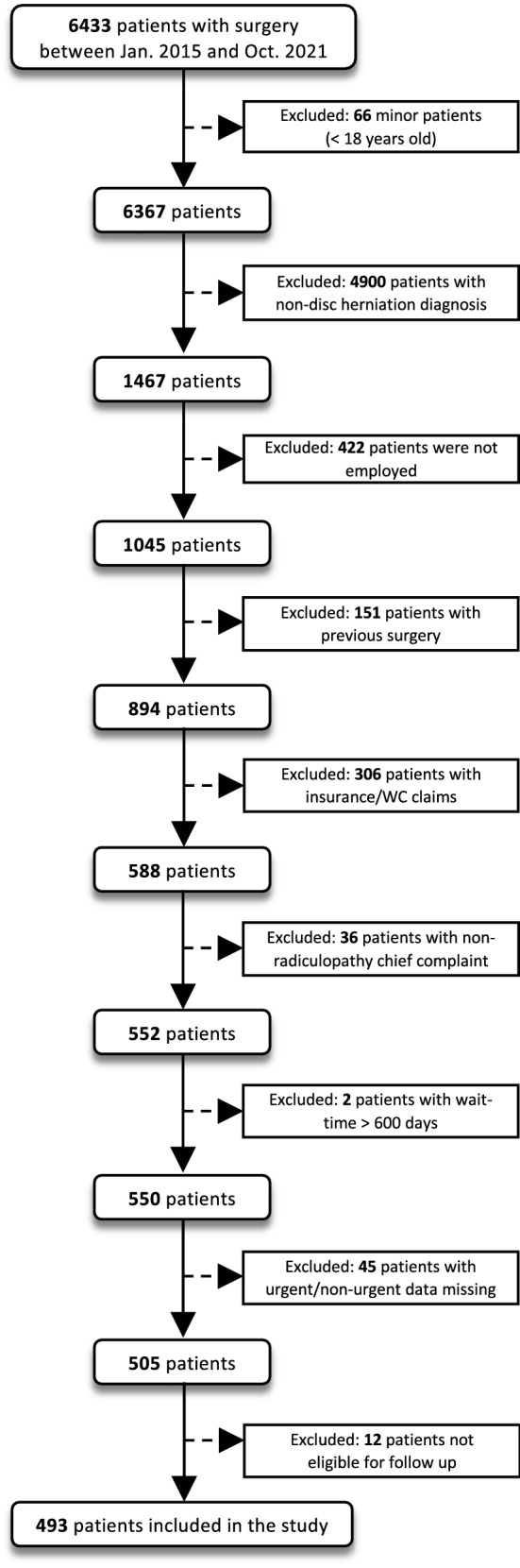
Flowchart of patient inclusion.

A total of 493 patients were included in this study (Fig. 1); 272 patients (55.2%) were in the short wait group, and the remaining 221 (44.8%) were long wait (Table 2). Both groups were similar in terms of sex, age, marital status, living status, education level, exercise, medications, and smoking status. The long wait group had a higher mean number of comorbidities per patient and a higher proportion with symptoms for 2 years or more. For the short wait group, the mean time from enrollment to surgery was 26.9 days (SD 16.7, median 26.0, IQR 27.5, range 0–59); in the long wait group, the mean was 144.4 days (SD 88.3, median 115.0, IQR 88.0, range 60–563).

**Table 2:** Patient characteristics.

	Short wait < 60 days n = 272 (55.2%)	Long wait > = 60 days n = 221 (44.8%)	p value
Patient variables			
Age (mean, SD)	41.9 (10.9)	43.0 (11.7)	0.249
Male (n, %)	137 (50.4)	128 (57.9)	0.094
Number of comorbidities (mean, SD)	1.7 (1.4)	2.1 (1.5)	**0.013**
BMI (mean, SD)	28.5 (6.0)	28.8 (6.7)	0.630
Single (n, %)	71 (26.4)	57 (25.9)	0.903
Living alone (n, %)	28 (10.4)	24 (11.0)	0.831
Education ≤ high school (n, %)	70 (26.1)	69 (32.2)	0.140
Active worker claim (n, %)	149 (56.7)	128 (59.5)	0.526
Medication use (n, %)	246 (91.1)	196 (88.7)	0.373
Symptom duration > 2 years (n, %)	79 (34.1)	95 (57.6)	** < 0.001**

Significant values are in [bold].

### Return to work

**Figure 2:**
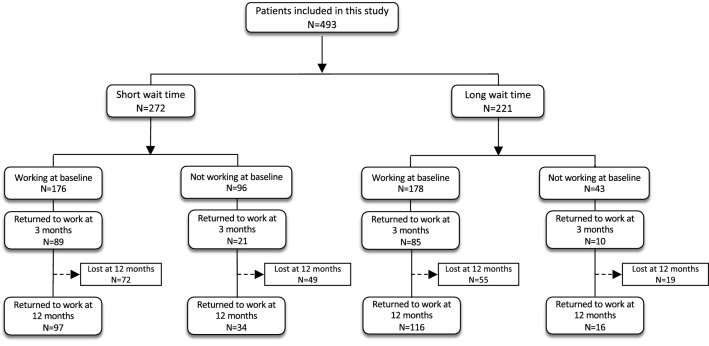
Working status flowchart.

Three-hundred and fifty-four patients (71.8%) were working at baseline with 176 patients (49.7%) in the short wait group and 178 patients (50.3%) in the long wait group. One hundred and thirty-nine patients (28.2%) were employed but not working at baseline (Fig. 2). All patients had return to work date at 3-months available as predefined as an inclusion criterion in the study. In the short wait group, 121 (44%) patients were lost to follow-up and did not have return to work date available at 12 months. In the long wait group, 74 patients (33%) were lost to follow-up and did not have return to work data available at 12 months.

Overall, in working patients, 174 (49.2%) returned to work at 3-month follow-up; at 12 months, 213 of 230 (93.8%) patients had returned to work. The proportions of patients who returned to work who were working at baseline did not significantly differ between groups at 3 months (50.6% vs 47.8%, p < 0.596) or at 12 months (93.4% vs 94.2%, p < 0.798).

**Figure 3:**
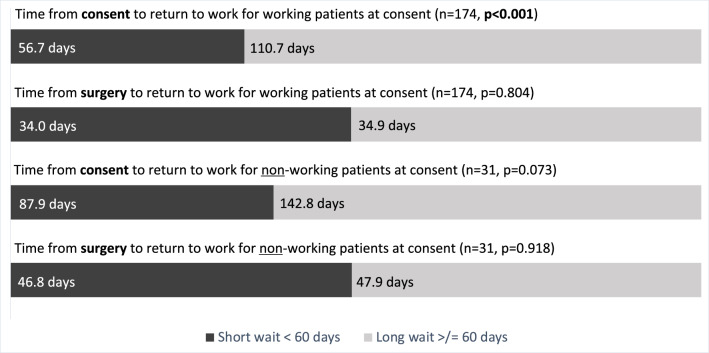
Time to return to work.

The working long wait time patients had a significantly greater mean time from baseline to return to work [110.7 days (SD 72.8) vs 56.7 days (SD 43.8], p < 0.001] (Fig. 3). Both groups returned to work at similar times after surgery [34.0 days (SD 22.9) vs 34.9 days (SD 21.4), p < 0.804]. Considering productivity loss, a longer wait from baseline to surgery corresponded to an average of $11,753.10 additional per long wait patient.

Within the overall 139 non-working patients at baseline, more had a shorter wait time (short wait: 96 patients (69.1%), long wait: 43 patients (30.9%). Thirty-one non-working patients at baseline (22.3%) returned to work at 3-month follow-up. Fifty patients (70.4%) returned to work at 12 months. The proportions of non-working patients at baseline who returned to work did not significantly differ between the groups at 3 months (21.9% vs 23.3%, p < 0.857) or at 12 months (72.3% vs 66.7%, p < 0.620).

In the 31 non-working patients who returned to work, the longer wait time group had a trend towards longer time from baseline to return to work [87.9 days (SD 83.6) vs 142.8 days (SD 59.3), p < 0.073]. The time from surgery to return to work date was similar between the wait time groups [46.8 days (SD 26.6) vs 47.9 days (SD 29.4), p < 0.918].

### Healthcare utilization from baseline (consent) to 3 months follow-up

**Figure 4:**
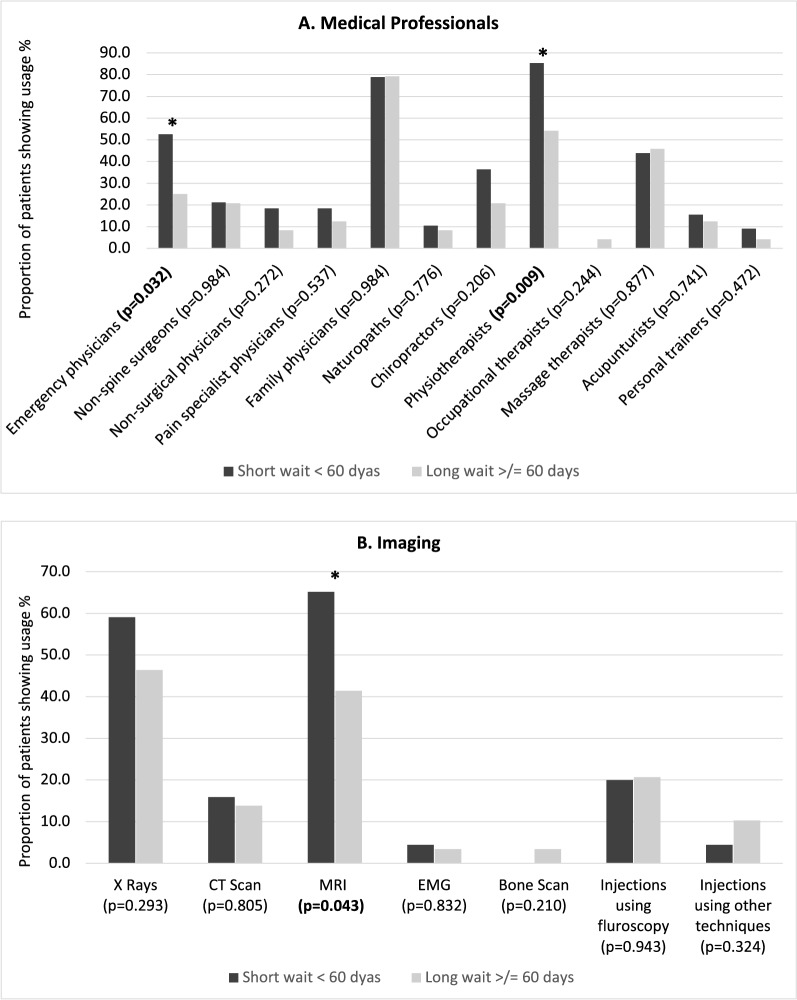
Healthcare utilization at 3 months.

Higher proportions in the short wait group visited the emergency department (52.6% vs 25.0%, p < 0.032), used physiotherapy (84.6% vs 72.0%, p < 0.001) and/or received an MRI (65.2% vs 41.4%, p < 0.043) (Fig. 4). Both groups were similar in terms of usage of nonsurgical physicians, pain specialists, family physicians, occupational therapist, naturopaths, chiropractors, massage therapists, acupuncturists and/or personal trainers, use of X rays, CT scan, EMG, bone scan usage and/or injections. The short wait time group had an additional healthcare usage of $518.21 direct costs per patient.

### Patient reported outcomes measures

Baseline preoperative unadjusted PROMs showed that the short wait group had higher mean NRS leg [7.81 (SD 1.9) vs 7.19 (SD 2.02), p < 0.001], ODI [50.94 (SD 16.6) vs 42.9 (SD 13.4), p < 0.001], EQ5D [0.4 (SD 0.2) vs 0.6 (SD 0.2), p < 0.001] and PCS [30.4 (SD 7.5) vs 31.6 (SD 7.6), p = 0.044] (Table 3). The mean change in PROMs at three-months follow-up from date of consent (baseline) were adjusted for baseline score. Only mean ODI and MCS had a larger change in the short wait group. Overall, at 3 months, a higher proportion of patients reached MCID in the short wait group for NRS leg pain (84.0% vs 75.9%, p < 0.040), ODI (78.1% vs 60.3%, p < 0.001), EQ-5D (48.2% vs 27.0%, p < 0.001), PCS (69.1% vs 58.4%, p < 0.036), and MCS (62.2% vs 50.0%, p = 0.020). Reaching MCID for NRS back pain was not associated with wait time.

**Table 3:** Patient reported outcomes.

	Short wait < 60 days	Long wait ≥ 60 days	p value
Unadjusted baseline variables			
NRS back (mean, SD)	6.34 (2.78)	6.16 (2.30)	0.429
NRS leg (mean, SD)	7.81 (1.85)	7.19 (2.02)	** < 0.001**
ODI (mean, SD)	50.94(16.60)	42.85(13.44)	** < 0.001**
EQ5D (mean, SD)	0.445 (0.223)	0.551 (0.193)	** < 0.001**
PCS (mean, SD)	30.43(7.51)	31.60(7.61)	**0.044**
MCS (mean, SD)	40.98(11.31)	42.56(11.20)	0.062
Adjusted** change scores from baseline to 3 months			
NRS back (mean, SE)	3.865 (0.138)	3.544 (0.151)	0.117
NRS leg (mean, SE)	5.231 (0.166)	4.937 (0.182)	0.234
ODI (mean, SE)	28.669 (1.098)	23.566 (1.232)	**0.002**
EQ5D (mean, SE)	0.295 (0.011)	0.266 (0.012)	0.073
PCS (mean, SE)	12.507 (0.662)	10.905 (0.733)	0.106
MCS (mean, SE)	9.832 (0.641)	7.118 (0.708)	**0.005**
Minimal clinical important difference (MCID) for patient reported outcomes			
MCID NRS back (n, %)	156 (69.6)	116 (62.0)	0.129
MCID NRS leg (n, %)	189 (84.0)	142 (75.9)	**0.040**
MCID ODI (n, %)	185 (78.1)	120 (60.3)	** < 0.001**
MCID EQ-5D (n, %)	107 (48.2)	50 (27.0)	** < 0.001**
MCID PCS (n, %)	150 (69.1)	104 (58.4)	**0.036**
MCID MCS (n, %)	135 (62.2)	89 (50.0)	**0.020**

*SD* standard deviation, *SE *standard error.

**Adjusted for baseline score.

Significant values are in [bold].

## Discussion

In this cost-consequence analysis of an observational cohort of patients receiving lumbar discectomy, obtaining early surgery was associated with an estimated cost savings of $11,234.89 per patient. The cost saving resided mostly in a decreased absenteeism from work with a short wait for surgery. For working patients at consent (baseline), the short wait time group had significantly shorter absence from work overall. For the employed but non-working patients, wait time did not affect the duration of absence from work overall. The short surgical wait group was associated with worse baseline pain scores, which potentially explained the increased healthcare usage and shorter surgical wait time, indicating they may have been preferentially treated. Secondarily, the higher proportion of short wait patients reaching the MCID for several patient reported outcomes, notably NRS leg pain, at 3 months can be explained by worse baseline pain scores with more room for improvement and less chronicity in symptoms.

Our study showed that time from surgery to return to work did not differ between short wait and long wait groups, irrespective of whether patients were actively working at baseline. Return to work was generally longer from baseline in the long wait group meaning the difference in loss of productivity resided mainly in the time off work while waiting. Once patients have received their surgery, patients return to work at the same rate and same time regardless how long they waited for surgery. A non-working but employed patient at baseline seemed to be twice as likely to be in the short wait group (< 60 days: 96 patients (69.1%), > / = 60 days: 43 patients (30.9%), compared to the working patient at baseline who were equally distributed between groups. This may be related to the more severe pain and frequent hospital visits and investigations preventing the patient to work and the incentive to do surgery early for those patients for symptom relief.

The short wait time group had an additional $518.21 of direct costs per patient, which was related to more emergency department visits, physiotherapy sessions and additional MRI. These patients had worse symptoms at baseline which likely explains the higher healthcare utilization and expedited treatment. Rampersaud et al. identified that a significant burden on the Ontario health-care system was attributed to spinal conditions, specifically targeting the cost associated with emergency department visits as potential for improvement^24^. The short wait group visited the ED more commonly and received more diagnostic imaging tests. Hence, it may be possible that expediting surgical management for patients that show increased healthcare utilization is beneficial to lessen the burden on the healthcare system. This could be explored in a future cost-effectiveness study.

The short wait for surgery had a greater proportion of patients reaching the MCID for many patient reported outcomes including NRS leg at 3 months post-operatively. This likely is explained by worse baseline pain scores creating more room for improvement as well as the acute nature and shorter duration of symptoms compared to the long wait group. The worse quality of life scores for the long wait group such as EQ-5D can be explained by the long wait group having more comorbidities, which is likely captured the tools that assesses overall health. A cost-effectiveness analysis from Finland showed that the number of quality adjusted life years (QALY) produced by discectomy within 60 days was greater than discectomy after 60 days (0.08 vs 0.05)^19^. Additionally, the cost per QALY for surgery within 60 days was approximately 40% less than the patients who received surgery after 60 days (1351 euros vs 2182 euros). It is possible that the cost saving per QALY would have been even greater for early surgery if indirect costs would have been considered. This is supported by the fact that most of the cost saving resides in indirect costs with loss of work productivity^16,17^. Patients in this study have not been randomized to create comparability of comparison. However, our findings are similar to other studies that have reported the outcome after lumbar disc herniation surgery to be better without undue treatment delay^4,20,28,32,33^. A systematic review assessed the optimal wait period for surgical intervention for lumbar disc herniation^34^. The overall outcomes and cost-effectiveness studies included in this review supported surgical intervention for lumbar radiculopathy within 8 weeks of symptom onset. Singh et al. revealed that the time to return to work is longer due to delayed surgery and symptom duration more than 2 years was a predictor of not returning to work^35^.

This study provides valuable insights into the economic repercussions of delayed surgery for disc herniation; however, there were some limitations. Group allocation was not randomized with each patient not having an equal chance of receiving one treatment or the other. Therefore, group differences occurred. However, this provided valuable information on potential factors that influence the timing of patients receiving early vs late surgery, which could be explored further in future studies. Patients with worse PROMs and utilizing more healthcare resources likely have been given preferential access to earlier surgery. The loss to follow-up at 12 months is potentially explained by the fact that most patients undergoing successful discectomy in Canada will be discharged to their primary care provider for long-term follow-up. The available data does not allow for separation of healthcare utilization after consent in the preoperative and postoperative periods. It is possible that the assumption that the difference in healthcare utilization residing mostly in the preoperative period is inaccurate. However, it is likely a reasonable assumption as it is supported by the similar time to return to work post-operatively in both groups. This study is a retrospective analysis of prospectively collected data. Individual wages are not part of the standard data collection. Therefore, it is possible that patient wages differ from the Canadian National average wage provided by Statistics Canada. Inherent limitations to the human capital approach are that it fails to account for the possibility that absent workers may be replaced, and thus the indirect cost is less when the perspective of the employer rather the employee is taken^25^. The ability to return to work might have less to do with the spinal disease and its treatment and more to do with the occupation, but it is unlikely that occupation varied significantly between groups. Missed workdays were calculated from consent to return to work. It is possible that the real time off work was underestimated given that time off work related to T1 wait times (from referral to consultation) was not assessed in this study. This report compared the economic impact of short or long T2 wait times (from consultation to surgery). Baseline (time of consent) was used as the start of work arrest. It is possible that time of work was overestimated in working patients with their work arrest starting at any time from baseline to surgical date. Furthermore, the data of reduced productivity (presenteism vs absenteeism) was not available^36^. Therefore, this study does not evaluate if returning to work was done with the same level of productivity or capacity.

## Conclusion

This cost-consequence analysis of an observational cohort showed decreased costs associated with early surgery of $11,234.89 per patient when compared to late surgery for lumbar disc herniation. From a socioeconomic perspective, early lumbar discectomy is cost beneficial and should be supported by the relevant health system payor. Secondarily, the higher healthcare utilization in patients receiving early surgery is potentially due to more severe and acute symptoms. This is counterbalanced by the additional productivity loss in the long wait group, which likely have a more chronic disease. Cost-effectiveness studies on this topic should include both direct and indirect costs because indirect costs are significantly influenced by surgical wait time.

## Data Availability

The datasets used and/or analysed during the current study available from the corresponding author on reasonable request.
